# Supplementary material for: Risk factors for sacrococcygeal pilonidal sinus: a systematic review and meta-analysis supplemented by genetic causal assessment
Source: Front Surg. 2026 Jan 7;12:1718589. doi: 10.3389/fsurg.2025.1718589 (PMC12819706; doi:10.3389/fsurg.2025.1718589)
Supplement: Supplementary file 2 [file Datasheet2.zip › Supplementary Data 2/MR_pipeline_p5e-8/forest_plots/ebi-a-GCST90018758.finngen_R12_L12_PILONIDALCYST.pdf]

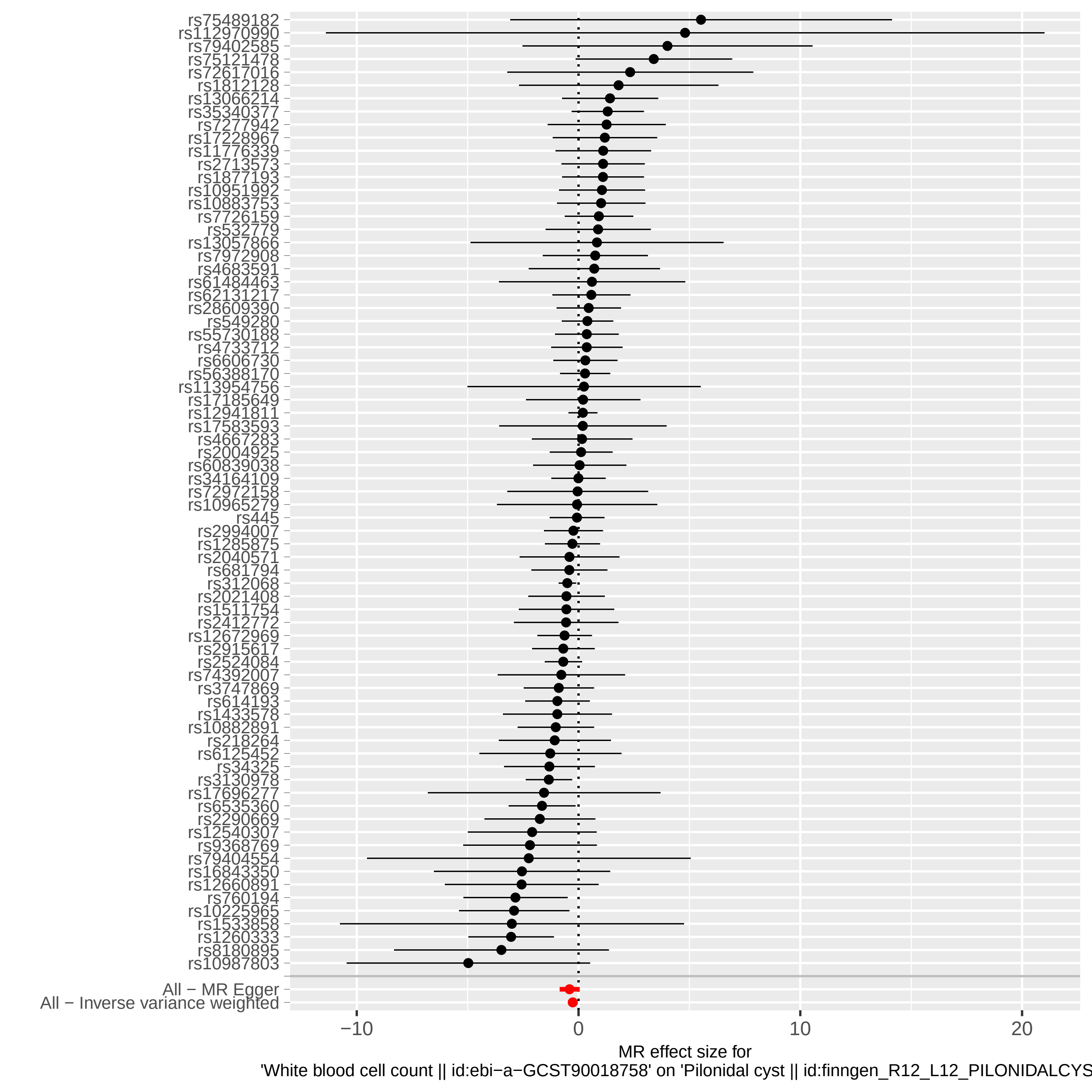
rs75489182
rs112970990
rs79402585
rs75121478
rs72617016
rs1812128
rs13066214
rs35340377
rs7277942
rs17228967
rs11776339
rs2713573
rs1877193
rs10951992
rs10883753
rs7726159
rs532779
rs13057866
rs7972908
rs4683591
rs61484463
rs62131217
rs28609390
rs549280
rs55730188
rs4733712
rs6606730
rs56388170
rs113954756
rs17185649
rs12941811
rs17583593
rs4667283
rs2004925
rs60839038
rs34164109
rs72972158
rs10965279
rs445
rs2994007
rs1285875
rs2040571
rs681794
rs312068
rs2021408
rs1511754
rs2412772
rs12672969
rs2915617
rs2524084
rs74392007
rs3747869
rs614193
rs1433578
rs10882891
rs218264
rs6125452
rs34325
rs3130978
rs17696277
rs6535360
rs2290669
rs12540307
rs9368769
rs79404554
rs16843350
rs12660891
rs760194
rs10225965
rs1533858
rs1260333
rs8180895
rs10987803
All − MR Egger
All − Inverse variance weighted
−10
0
10
20
MR effect size for
'White blood cell count || id:ebi−a−GCST90018758' on 'Pilonidal cyst || id:finngen_R12_L12_PILONIDALCYS
